# The diversification and potential function of microbiome in sediment-water interface of methane seeps in South China Sea

**DOI:** 10.3389/fmicb.2024.1287147

**Published:** 2024-02-06

**Authors:** Lulu Fu, Yanjun Liu, Minxiao Wang, Chao Lian, Lei Cao, Weicheng Wang, Yan Sun, Nan Wang, Chaolun Li

**Affiliations:** ^1^Center of Deep Sea Research and Key Laboratory of Marine Ecology and Environmental Sciences, Institute of Oceanology, Chinese Academy of Sciences, Qingdao, China; ^2^Laoshan Laboratory, Qingdao, China; ^3^State Key Laboratory of Mariculture Breeding, Key Laboratory of Marine Biotechnology of Fujian Province, Institute of Oceanology, College of Marine Sciences, Fujian Agriculture and Forestry University, Fuzhou, China; ^4^Center for Ocean Mega-Science, Chinese Academy of Sciences, Qingdao, China; ^5^South China Sea Institute of Oceanology, Chinese Academy of Sciences, Guangzhou, China; ^6^University of Chinese Academy of Sciences, Beijing, China

**Keywords:** cold seeps, sediment-water interface, methane metabolism, DAMO, oxic-hypoxic shifting

## Abstract

The sediment-water interfaces of cold seeps play important roles in nutrient transportation between seafloor and deep-water column. Microorganisms are the key actors of biogeochemical processes in this interface. However, the knowledge of the microbiome in this interface are limited. Here we studied the microbial diversity and potential metabolic functions by 16S rRNA gene amplicon sequencing at sediment-water interface of two active cold seeps in the northern slope of South China Sea, Lingshui and Site F cold seeps. The microbial diversity and potential functions in the two cold seeps are obviously different. The microbial diversity of Lingshui interface areas, is found to be relatively low. Microbes associated with methane consumption are enriched, possibly due to the large and continuous eruptions of methane fluids. Methane consumption is mainly mediated by aerobic oxidation and denitrifying anaerobic methane oxidation (DAMO). The microbial diversity in Site F is higher than Lingshui. Fluids from seepage of Site F are mitigated by methanotrophic bacteria at the cyclical oxic-hypoxic fluctuating interface where intense redox cycling of carbon, sulfur, and nitrogen compounds occurs. The primary modes of microbial methane consumption are aerobic methane oxidation, along with DAMO, sulfate-dependent anaerobic methane oxidation (SAMO). To sum up, anaerobic oxidation of methane (AOM) may be underestimated in cold seep interface microenvironments. Our findings highlight the significance of AOM and interdependence between microorganisms and their environments in the interface microenvironments, providing insights into the biogeochemical processes that govern these unique ecological systems.

## Introduction

Cold seeps are deep-sea extreme environments where deeply sourced methane-rich geofluids discharge at the seafloor and are widespread along continental margins (Joye and Kleindienst, 2017; Joye, 2020). Methane is the primary energy source supporting the macro- or microbial community surrounding the cold seep ecosystems (Sibuet and Olu, 1998). The biological activity of methane-oxidizing microorganisms in subsurface sediments and the water column considerably reduces the amount of methane that reaches the atmosphere (Valentine, 2011; Pratscher et al., 2018; Yang et al., 2020). The anaerobic oxidation of methane (AOM) is a key biogeochemical process regulating methane emission in anoxic subsurface sediment and is mediated by anaerobic methane oxidizing archaea (ANME) and sulfate-reducing bacteria (SRB) (Valentine, 2011; Pratscher et al., 2018; Yang et al., 2020). While aerobic oxidation of methane (AeOM) is mediated by type I aerobic methanotrophers affiliated with Gammaproteobacteria in aerobic or hypoxia water or the surface of marine sediments (Schubert et al., 2006; Steinsdottir et al., 2022).

Whether anaerobic or aerobic oxidation of methane dominates the methane consumption at the sediment-water interface depends on the supply of oxygen from bottom waters, which in turn dependent on bottom-water currents, the irrigation of the seafloor by animals, and the speed of upward fluid flow (Steinsdottir et al., 2022). Cold seeps are heterogeneous environments and create distinct surficial expressions and habitats, including carbonate pavement, microbial mats, and mussel beds (Du et al., 2022). Subtle differences existed in biogeochemical processes and velocity of upward fluids across these regimes. Carbonate pavement usually with the low methane fluids and hosted methane-oxidizing microbial community and carried out AOM predominantly. Microbial mat distributed surrounding the methane and sulfide-rich seeps, supporting a variety of thiotrophic bacteria, affiliated with Campylobacterota, which oxidized sulfide coupled to oxygen (Xu et al., 2022; Sun et al., 2023). Mussel beds distributed correlatedly to methane concentration, aggregating around methane seepage and gas vents (Cordes et al., 2009). Bioturbation and sulfide-rich upward fluids result in the co-occurrence of AeOM and sulfide oxidation in the chemosynthetic community. The biogeochemical processes are very diverse across the surface sediment of these habitats (Nunoura et al., 2016; Cui et al., 2019). However, limited information about the microbial diversity and functions in the sediment-water interface.

Sediment-water interface serve as the transitional region connecting deep water column to surface sediment, where intricate biological processes are shaped by the interplay of overlying water, eruptive fluids, and sediments (Zhuang et al., 2018, 2019c). The pronounced gradients in environmental conditions, such as methane concentration, along the interface, also create niche opportunities for microorganisms. Microbiomes play a vital role in material exchange and energy flow in interface layers between deep biosphere in sediments and upper water columns (Arakawa et al., 2006; Dang et al., 2010; Niu et al., 2017; Bhattarai et al., 2018; Wu et al., 2018; Cui et al., 2019; Chen et al., 2022). Sediment-water interface is the cyclical oxic-hypoxic shifting environments, and methane concentrations (ranging from 0.2 to 1 mmol/L) are negatively correlated with oxygen concentrations (ranging from 0.6 to 3.5 mg/L) (Xu et al., 2022). Microenvironments are developed not only by enormous plumes from the deep earth sphere but also by inherent background seawater, even the indigenous microbiomes in the original water environment (Joye, 2020). The microbiomes at the sediment-water interface have made significant contributions to methane microbial transformation, and methane serves as a vital source of matter and energy for the local biosphere (Boetius and Wenzhöfer, 2013; Wu et al., 2018; Jing et al., 2020). The main methane consumption in cold seep is aerobic oxidation (Fischer et al., 2012; Chakraborty et al., 2020), but owing to the limited sample collection equipment, there is a lack of a systematic microbial understanding of interface zones and comparison among these crucial interfaces.

To identify the diversity and potential function of microbiome at different habitats of sediment-water interface in cold seeps, we collected the deep water filtered samples from various velocity of methane fluid vents in two active cold seeps and microbial mat, mussel bed, and carbonate pavements in Site F, respectively. The main aims are to investigate: (i) Whether the microbial diversity is distinct at the sediment-water interface of different habitats in cold seeps (ii) how many ways of microbial methane oxidation including aerobic oxidation of methane, or DAMO, even SAMO, potentially occurred or co-occurred in the sediment-water interface of the different habitats of cold seep.

## Materials and methods

### Samples collection and geochemistry measurement

**FIGURE 1:**
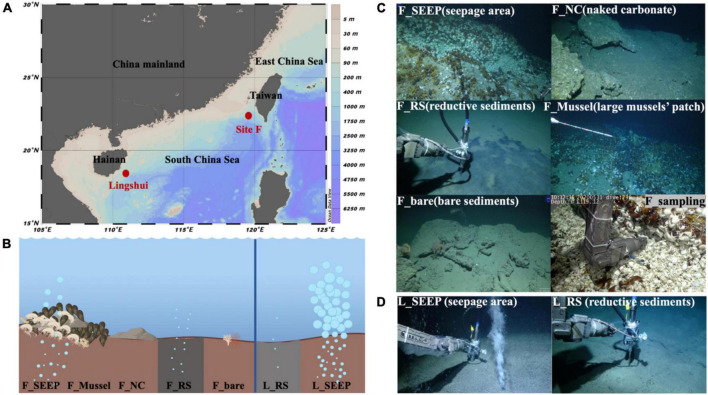
Sampling locations. **(A)** Site F and Lingshui cold seeps location in the South China Sea; **(B)** Conceptual graph of Site F and Lingshui representative microenvironment interface; **(C,D)** Pictures of sampling sites and organisms sampled during the present study. Representative photos of sampling habitats in Site F **(C)** and Lingshui **(D)**, respectively. All photographs were taken by ROV *Kexue*.

Seven deep water filtered samples were collected from the Site F and Lingshui cold seeps (Figure 1; Table 1) with the ROV *Faxian* deployed from the R/V *Kexue* during a research cruise on May 17th-June 19th, 2020, and May 22nd-June 23rd, 2021. The Site F cold seep is located southwest of Taiwan in the South China Sea. This site has clear micro-geographical differences with a steady intermittently eruptive situation. The Lingshui, a newly formed cold seep, is located in the southeast of Hainan. The seven sites represent different habitats from Site F and Lingshui cold seeps (Figures 1C, D; Table 1). In Site F, F_SEEP is located in the active seepage covered with macrofaunal communities such as mussels (*Gigantidas platifrons*) and shrimps (*Shinkaia crosnieri*). Gas bubbles occasionally appeared in this place (Figure 1C). The deep water was filtered above from the venting seepage F_Mussel was distinguished by a dense coverage of mussels and located a few meters close to the seepage, with no bubbles presence (Cao et al., 2021; Wang et al., 2021). The deep water was filtered above the mussel beds. F_NC was located at the carbonate pavement on the seafloor where no faunal assemblages were observed. We filtered deep water samples at the rock-water interface. The F_RS represented reduced black sediments, which were intermittently distributed at the edges of carbonate pavements or at a short distance from the rock. The filtered sample was collected from the sediment-water interface at black reduced sediment. Further away from the cold seep, the water sample was filtered at an area considered almost no methane leakage affected, called F_bare as control. In the Lingshui cold seep, the seep with exuberant bubbles, two samples were collected at the sediment-water interface of the seepage (L_SEEP) and reductive sediments (L_RS), respectively (Figure 1D).

**TABLE 1:** The sample location information.

Cold seep	Location	Water Depth (m)	Habitats
Site F (Figure 1C)	F_SEEP (seepage area)	∼1,150	Occasional gas bubbles occurred from the macrofauna; with many shrimps and/or crabs, little mussels
	F_RS (reductive sediments)	∼1,200	Reductive sediments
	F_Mussel (large mussels’ patch)	∼1,145	Large mussels’ patch (*Gigantidas platifrons*), little shrimps and/or crabs
	F_NC (naked carbonate)	∼1,150	Naked authigenic carbonates platform (without macrobiome)
	F_bare (bare sediments)	∼1,250	bare sediments
Lingshui (Figure 1D)	L_SEEP (seepage area)	∼1,800	Mud volcano, large quantity of gas bubbles seep
	L_RS (reductive sediments)	∼1,800	Reductive sediments

Sample at each habitat interface (uppermost 5 cm of the overlying water above the sediments) (Figure 1C F_sampling) was filtered 100 L deep sea water through a 0.22 μm polyethersulfone (PES) filter membrane by large-volume *in-situ* filtration equipment (LIFE) (Supplementary Figure 1, an experimental instrument designed by our group). All samples were immediately kept in liquid nitrogen, when they were retrieved on broad (<3 h).

Temperature, dissolved oxygen sensor (DO), CH_4_, and CO_2_ were *in situ* determined by a set of active sensor components assembled on the ROV *Faxian* (Cao et al., 2021). The concentration of NO_3_^–^, NO_2_^–^ and NH_4_^+^ were determined with the standard colorimetric methods using a continuous flow analyzer (Auto-Analyzer 3, Seal Analytical Ltd., UK) in the lab (Murphy and Riley, 1962; Catalano, 1987). Detection limits for NO_3_^–^, NO_2_^–^, and NH_4_^+^ were 0.02, 0.01, and 0.04 μmol L^–1^, respectively. The analytical uncertainty for all dissolved nutrients in replicate samples was <5%.

### DNA extraction and library preparation

Total genomic DNA from filters was extracted using the DNeasy PowerWater Kit (Qiagen, Germany), according to the manufacturer’s instructions with a little optimization. In the first step, the garnet grinding beads were changed with sterile chamilia beads; and vortex time at maximum speed was properly extended to 15∼20 min in the shocking step. After the extraction, DNA was pooled together based on the sample type, and stored at −80°C for further analyses. For archaea and bacteria 16S rRNA genes, different primer set procedures and protocols were used as previously (details in Supplementary Table 1; Fu et al., 2020). The library was sequenced on an Illumina NovaSeq platform and 250 bp paired-end reads were generated at Novogene Bio-Pharm Technology Co., Ltd., Beijing, China.

### Bioinformatic analysis

Paired end reads were demultiplexed based on their unique barcodes. The paired-end reads were then merged by using some of the read overlap generated from the opposite end of the same DNA fragment with FLASH (VI.2.7^1^). The merged reads were subsequently subjected to quality-based filtering with QIIME (Caporaso et al., 2010). Quality filtering on the raw tags was performed under specific filtering conditions to obtain the high-quality clean tag according to the FASTP. The tags were compared with the reference database (Silva database^2^) using UCHIME algorithm (UCHIME Algorithm^3^) to detect chimera sequences, and then the chimera sequences were removed. Then the effective tags were finally obtained. The remaining sequences were clustered into operational taxonomic units (OTUs) using Usearch (Version 7.0^4^) at 97% similarity levels, and the representative sequences were chosen based on the most common sequences. After randomly reducing the number of reads to the lowest number of reads in any individual sample, α and β diversity were measured by QIIME based on processed data. The richness indices (Chao1 estimates), diversity indices (Shannon index), and Good’s coverage were obtained. Non-metric multidimensional scaling (NMDS) ordination was conducted to distinguish the two sites based on the relative abundance of archaea and bacteria using the “Vegan” package in the R computing environment (R Core Team, 2014). Functional prediction of microbial community was carried out. Db-RDA (distance-based redundancy analysis) and permutest analysis were performed based on Bray-Curtis distances with the vegan package (2.4). The taxonomic information of the 16S rRNA gene was BLAST searched against the Functional Annotation of Prokaryotic Taxa (FAPROTAX) database (Louca et al., 2016). Co-occurrence networks were based on correlation coefficients and the cut-off was 0.75. Network properties and modularity were calculated with the igraph package. Network images were generated using Gephi^5^ (Bastian et al., 2009).

### Accession number of nucleotide sequences

All 16S-V3 and V4 sequences in this study were submitted to the NCBI Sequence Read Archive (SRA) database with BioProject ID PRJNA954131.^6^

## Results

### Geochemistry measurements

The water temperature of all sites was at 2.56∼3.69°C (Table 2), and the *in-situ* pH value was ∼7.9 (Supplementary Table 2). In Lingshui, amount of gas bubbles erupted from sediments (Figure 1D; Supplementary Figure 2). The concentration of methane in this region far exceeds 50 μM, the measurement range of the methane transducer equipped on ROV *Faxian* (the maximum is 50 μM). In Site F, the concentration of methane is approximately ∼8.1 μM at F_SEEP. The dissolved CH_4_ in F_Mussel was slightly lower compared to F_SEEP, ∼1.6 μM. Moving distance away from the seepage (F_SEEP), CH_4_ concentration sharply decreased. the concentration of methane at F_RS was approximately 0.055 μM. At the control site, F_bare, the concentration of methane was 0.33 μM. Nitrite ranged from 0.35 to 7.65 μM. At F_SEEP, the nitrite maximum concentration reached 7.65 μM, and was much higher than in F_bare. In contrast to nitrite, nitrate concentrations were 10.61∼30.2 μM. The concentration of nitrate in L_SEEP and L_RS was much higher than that of other sites. The concentration of ammonia had a broad range, from 1.15 to 4323.90 μM across all sites. The two highest ammonia concentrations were observed in F_SEEP and F_RS, where the concentration reached 105.37 and 4323.90 μM, respectively, while the ammonia concentration was closer to the other sites.

**TABLE 2:** Environmental characteristics of sediment-water interface of each habitat in two cold seeps.

Sampling location	L_SEEP	L_RS	F_SEEP	F_RS	F_NC	F_Mussel	F_bare
temp (°C)	2.56	2.56	3.59	3.63	3.65	3.69	3.22
CH_4_ (μM)	>50^*1^	NA	8.10	0.055	0.38	1.58	0.33
DO (mg/L)^*2^	2.4	NA	0.6–3.5	3.22	3.2	0.5–3.0	3.29
NO_2_^–^ (μM)	0.59	0.46	7.65	0.76	0.35	0.38	0.43
NO_3_^–^ (μM)	30.2	29.73	17.37	10.61	24.2	22.32	24.31
NH_4_^+^ (μM)	1.93	1.95	105.37	4323.90	1.15	3.37	2.65
SO_4_^2–^ (mM)^*3^	28	28	28	28	28	28	28

^*1^CH_4_ concentration in L_SEEP far exceeds the sensor measure range, 50 μM.

^*2^DO concentration in F_SEEP/Mussel has been previously reported by Xu et al. (2022).

^*3^take the high sulfate concentration (28 mM) in seawater as the environment background value, which makes it the most abundant and, often, important electron acceptor at cold seeps (Joye, 2020).

In Lingshui, dissolved oxygen (DO) in L_SEEP is approximately 2.4 mg/L as the ROV detected. In Site F, DO is in a cyclical oxic-hypoxic shifting state. Oxygen concentrations ranged from 0.6 to 3.5 mg/L in F_SEEP, and 0.5–3.0 mg/L in F_Mussel, while methane concentrations varied between 0.2 and 1 mmol. Methane concentrations were negatively correlated with oxygen concentrations (Cao et al., 2021; Xu et al., 2022). Temporal dynamics of methane and oxygen confirmed the presence of oxic/microoxic transitions that periodically occur inside the community. At other areas, DO is approximately 3.2 mg/L when the ROV traveled the Site F cold seep.

### The compositions of microbial communities

After quality control and filtration of chimeras and singletons, archaeal and bacterial reads were rarefied to 32,288 and 33,851 for each sample, respectively. All samples yielded 97∼284 and 275∼880 OTUs at a 97% sequence similarity level for archaea and bacteria, respectively. The Good’s coverage values range from 99.8 to 100% across all samples, indicating that sequences generated from these samples represented most of the microbial community (Supplementary Table 3). L_RS represents the highest diversity of archaea. F_RS and F_Mussel were associated with the highest archaeal and bacterial diversity indexes, respectively. Archaeal Chao1 richness ranged from 110.00 to 311.76 and the Shannon Index varied between 3.57 and 4.89 (Supplementary Table 3). And bacterial diversity index, Chao1 and Shannon were 275.00∼1123.50 and 5.48∼7.15, respectively (Supplementary Table 3). Overall, L_RS represents the lowest diversity and highest uniformity of archaea. F_RS and F_SEEP are associated with the highest archaeal and bacterial richness, whereas L_RS and F_bare the highest archaeal and bacterial evenness, respectively.

**FIGURE 2:**
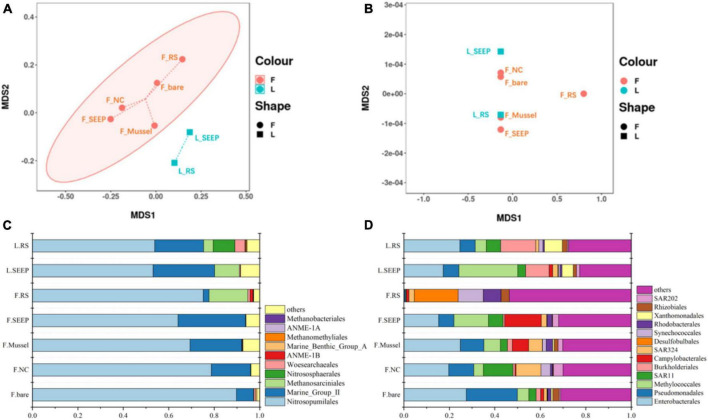
The NMDS analysis of archaeal **(A)** and bacterial **(B)** community and the compositions of archaeal **(C)** and bacterial **(D)** community in the order level in Site F and Lingshui cold seeps.

The NMDS plots showed significant differences in archaeal community composition across sites of Lingshui and Site F methane seeps (Figures 2A, B). While bacterial community composition was not distinctly different across all sites. Nitrosopumilales, Marine Group II (*Candidatus* Posidoniales), and Methanosarciniales were predominant archaeal orders across all samples. The relative abundance of Methanosarciniales was pretty high in two samples from the Lingshui methane seep (Figure 2C). In Site F, Nitrosopumilales and Marine Group II (*Candidatus* Posidoniales) were in predominant order, with increasing abundance of Marine Group II (*Candidatus* Posidoniales) and decreasing abundance of Nitrosopumilales along the concentration of methane decreasing (Figure 2C). Besides, Halobacterota was also highly abundant phylum in F_RS, accounted for 18.52% (Supplementary Figure 3A). Methanosarcina is a particularly dominant phylum in communities (Supplementary Figure 3B). Two predominant archaeal orders, Nitrosopumilales, and Marine Group II had different profiles along the methane concentration (Figure 2D). The relative abundance of Nitrosopumilales increased along the methane concentration, while Marine Group II was the opposite. In bacterial groups, Proteobacteria were the predominant bacterial phylum, with Alpha and Gammaproteobacteria being major classes, accounting for 30∼80% of the total bacterial community no matter in Lingshui or Site F (Supplementary Figure 4). In Lingshui, Enterobacterales, Pseudomonadales, Methylococcales, SAR11, Burkholderiales, and Xanthomonadales were the main orders. At Site F, there were some differences among the samples. In F_SEEP/Mussel/NC/bare, Enterobacterales, Pseudomonadales, Methylococcales, SAR11, Burkholderiales, SAR324, and Rhodobacterales were dominant groups. At F_RS, these orders were in low abundance, whereas Desulfobulbales were in the highest abundance order (Figure 2D). Besides, Campylobacterota were in high abundance of total bacterial groups in F_SEEP and F_Mussel.

**FIGURE 3:**
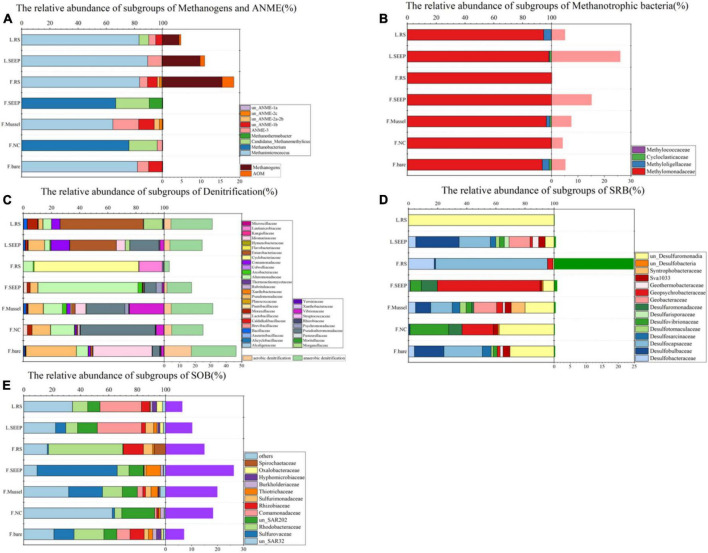
Community compositions at family level across different microenvironments at Lingshui and Site F. **(A)** Relative abundances of subgroups of methanogens and ANME. The orange bars on the right show the total percentages of methane-metabolizing archaea accounted for the total archaea. **(B)** Relative abundances of the subgroups of methanotrophic bacteria. The pink bars next to the barchart on the right show the methanotrophic percentages accounted for total bacteria. **(C)** Relative abundances of the dominant subgroups of putative denitrifying bacteria. The light orange/green bars on the right show the total percentages of putative aerobic/anaerobic denitrifiers accounted for total bacteria. **(D)** Relative abundances of the dominant subgroups of putative sulfate-reducing bacteria. The right viridis bars show the total percentages of SRB accounted for total bacteria. **(E)** Relative abundances of the dominant subgroups of putative sulfur-oxidizing bacteria (SOB). The right purple bars show the SOB percentages accounted for total bacteria. In all figures, F and L indicate the Site F and Lingshui.

Aerobic methane-oxidizing bacteria were observed in most of the sites and high abundance in seepage sites F_SEEP and L_SEEP, indicating that aerobic methane oxidation was the primary process. In contrast to AeOM, the anaerobic methane metabolizing microbes, ANME, and methanogens were in high abundance at F_SEEP, L_SEEP, and L_RS, while ANME had the highest abundance in F_RS. Correspondingly, Desulfocapsaceae, sulfate-reducing bacteria (SRB), the bacterial partner of AOM, was the dominant bacterial group in F_RS (Figure 3D). SAR324 and Sulfurovaceae were sulfur oxidizing bacteria (SOB) found across all sites (Figure 3E). SAR324 was much higher abundance than that of Sulfurovaceae across all samples except F_RS. Denitrifiers including Pseudomonadaceae and Moraxellaceae were present in all sites, with the lowest abundance in F_RS (Figure 3C).

**FIGURE 4:**
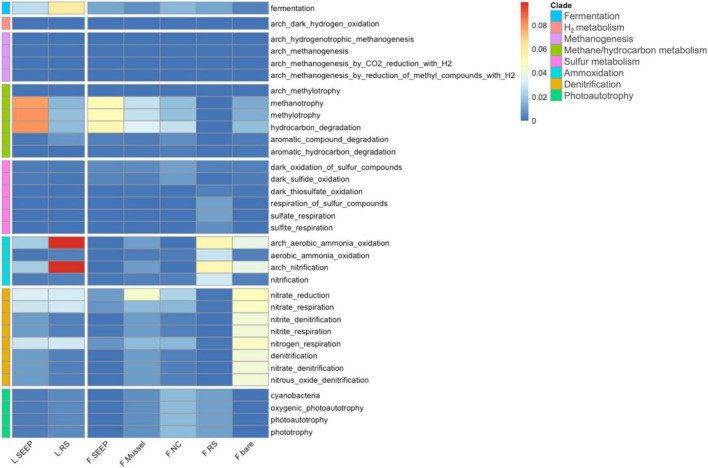
The heatmap of functional predictions of archaea and bacteria in sediment-water interface of Lingshui and Site F cold seeps.

Methanogens and ANME are more abundant in bare sediments, while aerobic methane-oxidizing bacteria are more prevalent in almost all other detected areas, except for F_RS (Figure 3A). This suggests that the processes of AeOM and AOM are more intense in L_SEEP/RS and F_RS. Particularly in F_RS, methane metabolism is dominated by methane production and AOM. In other areas (F_SEEP/Mussel/NC/bare), the main methane metabolic process is aerobic methane oxidation (Figures 3B, 4). The abundance trends of denitrifiers and sulfate-reducing bacteria (SRB) exhibit an opposite pattern. Anaerobic denitrifiers are present in all areas, with the lowest abundance in F_RS, while SRB is only detected in F_RS with a high relative abundance (Figures 3C, D). Sulfur-oxidizing bacteria (SOB) are more abundant in Site F compared to Lingshui (Figure 3E). Both aerobic and anaerobic oxidation processes of methane are relatively strong in Lingshui, with anaerobic methane oxidation dominated by DAMO. In Site F, DAMO is detected in F_SEEP/Mussel/NC/bare, with the highest degree of anaerobism observed in F_RS, where the anaerobic oxidation of methane is mainly carried out by SAMO. Sulfur oxidation in Site F is generally higher than that in Lingshui. This can be attributed to the more reductive conditions and higher degree of organic matter degradation in Site F.

### Prediction of potential microbial metabolic pathways in two methane seeps

The potential metabolic pathways demonstrated that microorganisms involving methane oxidation and hydrocarbon degradation were the most highly abundant across all samples (Figure 4). L_SEEP and F_SEEP had the most abundant bacterial groups involved in AeOM and hydrocarbon degradation, while L_RS and F_RS were low abundant. F_Mussel and F_NC were also observed as bacterial groups involving AeOM and hydrocarbon degradation, but lower than these in seepage sites. For the sulfur cycle, a high abundance of microbes involving sulfur, sulfide, and thiosulfate oxidation and respiration of sulfate and sulfur compounds were in F_RS. For the nitrogen cycle, the microbes involving nitrate reduction, and nitrate respiration were observed in most of the samples and aerobic ammonia oxidation were in high abundance across all samples. Interestingly, the microorganisms contained phototrophy and photosynthetic metabolic pathways were in F_RS.

### The effects of environmental factors on microbial diversity and co-occurrence analyses

**FIGURE 5:**
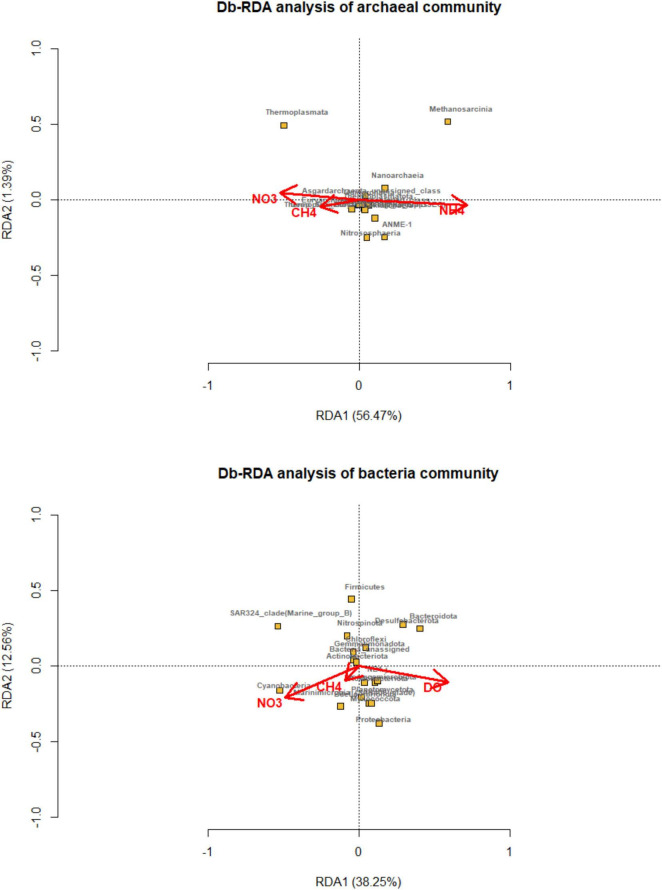
Distance-based redundancy analysis (dbRDA) plot of the forward selection based on environmental variables (methane, DIC, nitrite, nitrate, ammonia and dissolved oxygen) fitted to the taxonomic composition of bacterial and archaeal communities (at the class level). The significant explanatory variables are showed (red vectors). Microbial taxa are indicated by yellow squares and labeled with black words.

The environmental factors had an impact on the composition of microbial community. Distance-based redundancy analysis was carried out and showed that the methane, ammonia, and nitrate concentration main predictor variables, explained 56.47 and 1.39% of the total variation in archaeal compositions (Figure 5). Thermoplasmata and unassigned Euryarchaeota were correlated with methane and nitrate, while Methanosarcina and ANME were related with ammonia concentration. While, methane, nitrate, and dissolved oxygen were primary environmental factors that affected on bacterial community, explaining 38.5 and 12.56% of the total variation.

**FIGURE 6:**
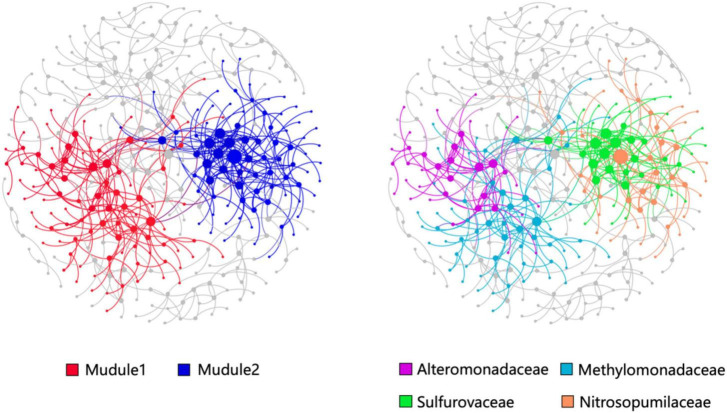
Co-occurrence networks and their topological features based on the total prokaryotic communities in the high-methane-supply, low-methane-supply, and control groups. The size of nodes (OTUs) is related to the relative sequence abundance of OTUs. Large modules with ≥10 nodes are shown in different colors, while the small modules are shown in gray color.

The OTUs with a relative abundance of more than 300 were constructed the co-occurrence network consisted of 148 nodes and 256 edges (Figure 6). Modularity analysis showed two high abundance modules in the network labeled in Figure 6. Module 1 was comprised of a group of OTUs that were phylogenetically close and belonged to the Methylomonadaceae and Alteromonadaceae clades. This finding suggests that AeOM could couple to denitrification in aerobic sediment-water interface. Module 2 was mainly composed of Sulfurovaceae OTUs and Nitrosopumilaceae OTUs, respectively. It indicated that Sulfurovaceae may mutualize with Nitrosopumilaceae, and sulfur oxidation and ammoxidation co-occur in the sediment-water interface of methane seeps.

## Discussion

### The composition of microbial community varied in different habitats of sediment-water interface of methane seeps

At Site F, the richness of the archaeal community of F_SEEP/NC was low, and at other areas was relatively high. A coincidence is that the archaeal community richness of higher methane concentration seepage/lush area is lower. There might be two reasons for this observation. Firstly, compared to F_RS, the interfacial layers of F_SEEP/NC, which contained compact carbonate rocks, have limited material and energy exchange with the underlying sediments. As a result, archaea present in the macrofaunal communities and seawater flowing through the sediment-water interface layers may be the main clades in this environment. Secondly, macrofaunal communities were selected as the exceptional environment for the archaeal community, which had a guiding selection and influence on the microbial community and may also have a certain impact on the diversity of the bacterial community (Xu et al., 2022). Other microenvironments, such as F_Mussel/RS/bare exhibit higher archaeal diversity and richness. This could be attributed to the material energy flows and exchange between surface sediments and bottom water. Notably, sediments-water interface samples were greatly influenced by sediments whose microbial diversity is much higher than that of seawater environment (unpublished data). The local environment at F_RS, characterized by micro seepage, seemed to be more favorable for the anaerobic oxidation of methane, and frequent exchange between substrate sediments and overlying water likely occurred. Consequently, microbial diversity in the interface layer of F_Mussel/RS/bare is much higher than F_SEEP/NC (Supplementary Table 3), where little material communication with surface sediments is observed. The presence of microregional and/or temporal hypoxic environments in cold seep has been previously reported at Site F by Xu et al. (2022). The distribution patterns of these interfaces align with the cores of the oxygen minimum zone in the central Mexican Pacific (Pajares et al., 2020). The distribution of the microbial diversity and the main clades changed along the transition from the oxygen-rich area to the OMZs, demonstrating the sensitivity of key bacterial groups to deoxygenation.

Bacterial communities exhibit a high level of complexity in interface microenvironments. The relative abundance of Methylococcales, responsible for aerobic methane oxidation, increases with the distance closer to the methane seepage/bio-flourishing area. Communities from F_RS displaying steady-state DIC-methane-sulfate dynamics contained higher abundances of several clades belonging to *Candidatus* Aminicenantota, Dehalococcoidia, Rhodobacterales, and Rhizobiales. Some were more abundant members of bacterioplankton in the water column but rare in sediments (Miksch et al., 2021), suggesting their widespread distribution in bottom boundary layers (Kubo et al., 2002). Consequently, DMS/DMSP transformation might be taking place, which is predominantly in SAR11 and Rhodobacteraceae in seawater, whereas they were mainly in alpha-proteobacterial Rhizobiales, Rhodobacterales as well as gamma-proteobacterial Pseudomonadales (Song et al., 2020). DMSP related groups of Pseudomonadales obtain energy from the oxidation of sulfur, thiosulfate, or both, while producing sulfate (Yu et al., 2023). Meanwhile, Rhodobacterales and Pseudomonadales also dominate the *nirS*-denitrification microbial community (Ai et al., 2017). SAR11, Pseudomonadales, Rhizobiales, and Rhodobacterales play vital roles in denitrification and DMS/DMSP production, which are important drivers of global sulfur cycling and can impact climate (Zhang et al., 2019). Besides, the phylum Chloroflexi and Cyanobacteria, as dominant clades in F_RS, have been reported to conduct an anoxygenic phototrophic lifestyle (Ward et al., 2019). Diverse phototrophic Chloroflexi members are distributed in deep-sea cold seep (Zheng et al., 2022). Additionally, Xanthomonadales has a dominant role in Lingshui and is known to degrade aromatic compounds and hydrocarbons as a preferred carbon and energy source, with the ability to utilize a variety of other carbon substrates (Saddler and Bradbury, 2005; Gutierrez, 2019). SAR202, more prevalent in Site F, may be likely to participate in the degradation of cyclic alkanes (Landry et al., 2017). SAR202 clade in dark-ocean bacterioplankton encoded multiple families of paralogous enzymes involved in carbon catabolism, including several families of oxidative enzymes that hypothesized participate in the degradation of cyclic alkanes, and organosulfur compounds metabolism. Many SAR202 members appear to be sulfite-oxidizers and are predicted to play a major role in sulfur turnover in the dark water column (Landry et al., 2017; Mehrshad et al., 2018).

Cold seep, particularly methane-rich areas and trace leakage zones are primarily dominated by carbon metabolism, with metabolic cycles of other generative elements relatively suppressed and restricted, such as nitrogen and sulfur (Sun et al., 2020; Xu et al., 2022). Microorganisms involved in the cycling of generative elements struggle to survive and maintain the balance of these elements within the cold seep ecosystem. Temporal dynamics of methane and oxygen concentrations confirm the periodic occurrence of oxic-hypoxic transitions at the interface scale and suggest the presence of diverse and widespread microbiomes adapted to these conditions. In aerobic environments, microorganisms degrade complex OM to produce carbon dioxide, and inorganic salts such as nitrate and sulfate serve as the main electron acceptors in anaerobic environments (D’Hondt et al., 2002). The microbial composition is largely shaped by the bottom microenvironment. Chemoautotrophic microbes (Campylobacterales, Desulfobulbales, some SAR324 members) and chemoheterotrophic clades (SAR11, some other SAR324 members) are detected in this study are not only found in cold seep but always occurred in dark ocean. Besides, heterotrophic microbe (Enterobacterales, Microtrichales, SAR202) are also detected, with high relative abundance (Figures 3, 6). These microorganisms are metabolically active and provide alternative sources of energy (Joye, 2020).

### Methane concentration or flux has an impact on the diversity and function of the microbiome

The diversity of microbiomes in interface microenvironments is strongly influenced by the eruptive fluids. The anaerobic methanogenesis and aerobic/anaerobic methanotrophic microbiomes co-exist in naked sediment areas, which are in line with CH_4_ emissions from cold seep in the interface microenvironments. The flux of geofluids, serving as a vector for the upward dispersal of deep biosphere microbes from subsurface to interface environments, shapes the microbiomes of interface microenvironments and provides a general mechanism for the maintenance of microbial diversity in the subseafloor deep biosphere (Chakraborty et al., 2020). And fluids diffuse to the surface through the pores in the sediments, methane (CH_4_) is oxidized to carbon dioxide in the anaerobic and sulfate-rich sediments under the combined action of methane anaerobic oxidizing bacteria and sulfate-reducing bacteria (Lever, 2011). Considering the naked sediment areas processing CH_4_ with a slight leak, there should be more anaerobic microbes in the sediment layers reaching the interface with the outward eruption/leakage of gas. In Lingshui, oxic and hypoxic methane-metabolizing microbes appear simultaneously in the same microenvironment (L_SEEP/RS), whereas in Site F, only the anoxic are detected, particularly in reductive sediments (F_RS). Additionally, the majority of methanotrophic microbiomes detected in the other microenvironments of Site F (F_SEEP/Mussel/NC/bare) are aerobic bacteria, with the highest abundance of methane oxidation bacteria found around the seepage area. These two cold seeps are developed in different stages, with the microecological region of Lingshui still forming, while Site F exhibits a more established and mature regionalization (Figure 6). In exposed reductive sediments, Methanosarciniales was the dominant group, regardless of whether in Site F or Lingshui (∼17.1% in F_RS, ∼10.9% in L_SEEP, ∼4.26% in L_RS). This suggests that more relative abundance of the methane metabolic and related microbiome occurs in Lingshui. Based on the previous description and supplementary figures (Supplementary Figure 2), there is a huge eruption of gaseous fluid in Lingshui, and occasionally appeared bubbles in Site F. The fluid seepage intensity is largely controlled by flow rate, thus more nutrients could dissolve in bottom water in relatively low flow rate conditions at leakage areas like F/L_RS, while nutrients migrate upward to shallow water due to high flow rate at seepage zones like F/L_SEEP (Li et al., 2021). In summary, the intense eruption in Lingshui is dominated by methane, along with long-chain hydrocarbons, even oil gas components, serving as fast-food feed for microbiomes, and the methane-related microbial community should be relatively simple. In Site F, the subsurface fluids happened contingently at the cyclical oxic-hypoxic fluctuating interface, where a zone of intense redox cycling of carbon, sulfur, and nitrogen compounds, methane and hydrocarbons emissions from seepage area are mitigated by not only methanotrophic bacteria, but related sulfur, and nitrogen metabolizing microorganism.

Anaerobic oxidation of methane via DAMO is an important metabolic approach that cannot be ignored in the oxic-hypoxic shifting microenvironments. Heterotrophic metabolism is the primary activity of microorganisms, with OM serving as an electron donor, while oxygen, sulfate, and nitrate act as electron acceptors, providing material and energy sources for microbial growth. The association of ANME-2d archaea and NC10 bacteria with the DAMO process has been proposed from the coastal mangrove wetland to the South China Sea sediments (Chen et al., 2022). Nitrite-dependent DAMO process is more important in the hydrate-bearing trough (Jiang et al., 2022). The relative abundance of Methylomonadaceae (o_Methylococcales) is highest in the methane seep/bio-luxuriance zone among these microenvironments, where the methane concentration can exceed 31,200 ppm (∼55.2 μM), and DO ranges from 0 to 3.3 mg/L. This suggests that these methane-oxidizing bacteria can survive in both hypoxic and anoxic conditions, potentially due to the presence of AOA (o_Nitrososphaerales). The content of Nitrososphaerales and Woesearchaeales in the boundary layer of methane seepage (L_SEEP) and reductive sediment (L_RS) area was obviously different. The relative abundance of Nitrososphaerales is higher in L_RS, whereas it is lower in F_RS. As previously reported, considering the transcription of the *nirK* gene in AOA detected by Lund et al. (2012), AOA are not only ammonia oxidizing but also denitrifying (Bartossek et al., 2010; Lund et al., 2012; Stieglmeier et al., 2014; Kozlowski et al., 2016). The results indicate the occurrence of aerobic and anaerobic methane oxidation coupled with denitrification, and possibly more complex carbon and nitrogen coupling processes (Figure 4). Previous studies have reported that aerobic methane oxidization is mainly conducted by type I methanotrophs in low-intensity seepage and carbonate rocked Site F, but also via nitrite-dependent anaerobic methane oxidation (also known as “nitrate/nitrite-dependent anaerobic methane oxidation,” or DAMO) process by Methanoperedens and Methylomirabilis in reductive sedimentary of the Haima seep and Xisha Trough, respectively (Jing et al., 2020). Meanwhile, it has been proved that the highest abundance of methane-oxidizing bacteria, Methylomonadaceae can potentially participate in AOM by accepting electrons for N-oxides reduction (Cabrol et al., 2020). Given the high relative abundance of these N-metabolic-dependent methane oxidizers, it is suggested that the previously overlooked DAMO process may serve as a crucial methane sink in both Site F and Lingshui (Figure 6). Moreover, Methylomonadaceae, including the predominant genus Methyloprofundus as well as Marine Methylotrophic Group 2, play major roles as anaerobic methane-oxidizing bacteria in interfaces, especially in F_RS, L_SEEP.

**FIGURE 7:**
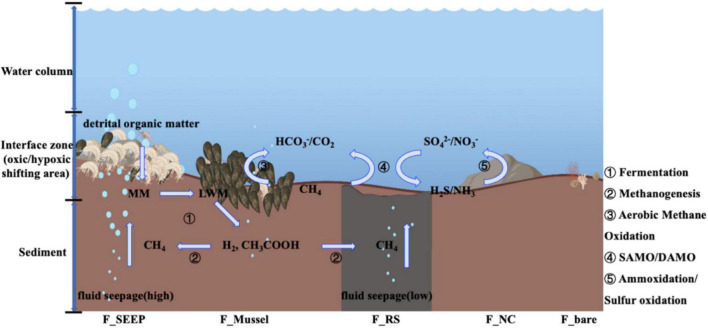
Web model coupled to carbon/sulfur/nitrogen metabolic process at the interface of Site F by the microbial 16S rRNA gene analyses [During exoenzymatic hydrolysis, detrital organic matter is buried at the interface as macromolecules (MM), then broken down to low molecular weight matters (LMW). Fermentation further degrades LMW to short chain fatty acids such as acetate, and hydrogen, which would act as substrates to produce methane (Zhuang et al., 2019a; Joye, 2020). Meanwhile, fluid seepage from the deep sediment layers delivers additional methane to the interface. At the cyclical oxic-hypoxic fluctuating interfaces, when shifting to aerobic state, methane oxidizing bacteria (dominant by Methylococcales) mediate the aerobic/anaerobic methane oxidation, while AOA mediate ammonia (NH_3_) oxidation to produce nitrate/nitrite and SOB mediate hydrogen sulfide (H_2_S) oxidation to produce sulfate, which provide the reaction substrates for anaerobic methane oxidation. When anaerobic, the anaerobic methane oxidation coupled with SAMO/DAMO produce carbon dioxide and reductive substances such as hydrogen sulfide/ammonia. The serial numbers represent different metabolic microbial groups. ➀ Fermentation microbiomes, mainly Flavobacteriales, Bacteroidales and so on (Zhuang et al., 2019a,b); ➁ Methanogens, mainly including *g_Candidatus Methanomethylicus, g_Methanobacterium, g_Methanimicrococcus* (Zhuang et al., 2019a; Joye, 2020); ➂ Aerobic methane oxidation microbiomes, such as Methylomonadaceae, Methyloligellaceae; while AOM microbiomes, not only ANMEs, maybe also including Methylomonadaceae; ➃ AOM coupled SAMO, the prevalent SRB microbiomes are Desulfocapsaceae, Geopsychrobacteraceae, Desulfobulbaceae; AOM coupled DAMO, the predominant aerobic denitrifiers are Pseudomonadaceae, and anaerobic denitrifiers are Alteromonadaceae, Pseudoalteromonadaceae; ➄ Ammonia-oxidizing microorganisms, including Nitrosopumilaceae, Nitrososphaeraceae and so on; SOB principally including Sulfurovaceae, Rhodobacteraceae].

Denitrification and sulfate reduction are key reduction processes coupled with anaerobic oxidation of biomethane (Figure 7). Both denitrifiers (aerobic and anaerobic included) and SRB are all detected, whereas the abundance of denitrifiers is much more than SRB, but F_RS. The reductive sediment at Site F has the highest degree of reduction with the lowest oxygen content. It has previously been reported that aerobic denitrification was carried out for complete nitrogen removal by *Achromobacter* (o_Burkholderiales; f_Alcaligenaceae), *Acinetobacter* (o_Pseudomonadales; f_Moraxellaceae) and *Pseudomonas* at DO in the range of 3∼10 mg⋅L^–1^ and *Bacillus* (o_Bacillales; f_Bacillaceae) carry out denitrification at a DO concentration of 3.93∼7.65 mg⋅L^–1^ (Zhu et al., 2013; Ji et al., 2015). Denitrification was observed for *Pseudomonas* (o_Pseudomonadales; f_Pseudomonadaceae) which co-respired using NO_3_ and O_2_ under controlled oxygen conditions (0∼8 mg/L) with ethanol and methanol serving as carbon sources (Gómez et al., 2002). Given higher abundance of anaerobic denitrifiers compared to their aerobic counterparts, it is possible that AOM occurs through a tightly interacting microbiome. This interaction may involve cryptic biogeochemical cycling, wherein aerobic MOB interacts with denitrifiers/SRB. Correlation between Methylomonadaceae and *Geobacter*, Desulfobulbaceae, indicates that AOM may occur through an interaction between methane oxidation bacteria and denitrifiers, iron-cycling partners (Fe-reducer), and SRB.

From a microbial-mediated geochemical perspective, methane aerobic oxidation and anaerobic oxidation coupled with denitrification, Fe-reduction, and sulfate reduction are two potential processes occurring in the overlying water environment. The interfacial layer is a special environment where the oxic-hypoxic state maintains a continuous transition. Alternatively, there could be a thin layer of anaerobic microenvironment covered by water on the interface layer under the aerobic while the sensor has just reached the aerobic/anaerobic surface position. By consuming O_2_ to nanomolar levels, aerobic nitrifying microbes cede their competitive advantage for scarce forms of N to anaerobic denitrifying bacteria, and anaerobes cannot sustain their own low-O_2_ niche, the physical O_2_ supply restores competitive advantage to aerobic populations, resetting the cycle (Penn et al., 2019). The resulting ecosystem oscillations induce a unique geochemical signature within the most reductive area, F_RS, maybe the oxygen-deficient zone, and short-lived spikes of ammonium that are found in measured profiles. In these microenvironments, oxygen could be rapidly consumed by microorganisms dwelling in the upper few millimeters of sediments or living symbiotically with animals at the sediment-water interface (Boetius and Wenzhöfer, 2013). Oxygen is also consumed by abiotic reactions that recycle reduced substrates (e.g., hydrogen sulfide). A great deal of oxygen consumption was due to the activity of SOB, thiotrophic and methanotrophic microorganisms, both free-living organisms and those living in symbiosis (Dubilier et al., 2008; Boetius and Wenzhöfer, 2013). Under anoxic conditions, the AOA clade can produce O_2_ for ammonia oxidation by itself while simultaneously reducing nitrite to nitrous oxide (N_2_O) and dinitrogen (N_2_) (Kraft et al., 2022; Martens-Habbena and Qin, 2022). The discovery of ammonia oxidation within the domain Archaea has substantially changed the understanding of the global nitrogen cycle (Kerou et al., 2021; Zhao et al., 2021; Martens-Habbena and Qin, 2022). Our results provide a possible explanation for the presence of AOA clade (o_Nitrosopumilales) in the oxic-hypoxic shifting microenvironments. These findings suggest that cold seep is rigorously controlled by extremely active carbon metabolism, especially the metabolism and transformation of methane. Nitrogen is constrained, which might have implications for the evolution of the carbon-nitrogen coupled cycle in chemoautotrophic ecosystems. Microbial ecosystem dynamics might give rise to variable ratios of denitrification to sulfate reduction, providing a mechanism for the unexplained variability of these pathways observed in cold seep.

## Conclusion

Methanotrophy is the primary fluids microbial transformation in bio/carbonates/sediments-water interface, a zone characterized by intense redox cycling of carbon, sulfur, and nitrogen compounds. Seeps exhibit high spatial heterogeneity and differ significantly from non-seep environments, especially at Site F. However, many critical issues remain regarding the partitioning of seep fluid and gas metabolism among the diverse organisms suspected to participate. This study emphasizes that the interface is a crucial observation zone shaped by overlying water, eruptive fluids, and sediments in the deep ocean floor. As a globally widespread hydrogeological phenomenon, more attention should be paid to bio-transcription, protein levels, and *in situ* tracking, which could reflect the actual life activities in future process(es).

In our study, the microbial functions predicted by the 16S-based taxonomy might be limited, metagenomics and macro transcriptome-based methods are needed. The microbial ecological roles in seepages require further investigation. Our future studies will focus on examining the potential interactions that contribute to microbial environmental adaptation, particularly in cyclical oxic-hypoxic fluctuating environments. Furthermore, metagenomics and macro transcription analysis combined with in situ culture experiments in these special interface zones are also given more attention in order to gain a deeper understanding of their microbial dynamics.

## Data availability statement

The datasets presented in this study can be found in online repositories. The names of the repository/repositories and accession number(s) can be found below: https://www.ncbi.nlm.nih.gov/, PRJNA954131.

## Author contributions

LF: Formal analysis, Investigation, Methodology, Writing—original draft, Writing—review and editing. YL: Writing—review and editing. MW: Writing—review and editing. CL: Investigation, Writing—review and editing. LC: Investigation, Writing—review and editing. WW: Formal analysis, Methodology, Software, Writing—review and editing. YS: Formal analysis, Methodology, Writing—review and editing. NW: Formal analysis, Methodology, Writing—review and editing. CLL: Conceptualization, Funding acquisition, Project administration, Resources, Writing—review and editing.

## Footnotes

1
http://ccb.jhu.edu/software/FLASH/

2
https://www.arb-silva.de/

3
http://www.drive5.com/usearch/manual/uchime_algo.html

4
http://drive5.com/uparse/

5
http://gephi.github.io/

6
https://www.ncbi.nlm.nih.gov/sra/PRJNA954131
