# Supplementary material for: The diversification and potential function of microbiome in sediment-water interface of methane seeps in South China Sea
Source: Front Microbiol. 2024 Feb 6;15:1287147. doi: 10.3389/fmicb.2024.1287147 (PMC10878133; doi:10.3389/fmicb.2024.1287147)
Supplement: Supplementary file 1 [file Data_Sheet_1.pdf]

The diversification and potential function of microbiome in sediment-water interface of methane seeps in South China Sea

Figures

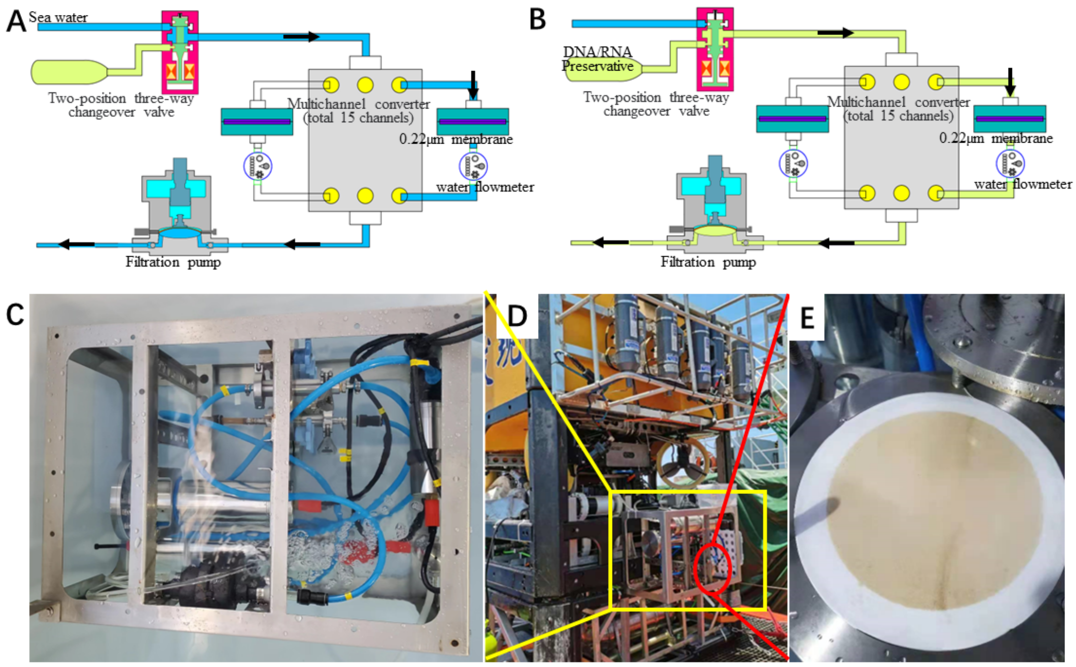

Fig. S1 Large-volume *In-situ* Filtration Equipment (LIFE) (an experimental instrument designed by our group). (A). Schematic structure and workflow diagram of LIFE apparatus. Seawater was pumped into the filtration chamber;

(B). DNA/RNA preservative (DNA extraction buffer or RNALater solution) was injected into the chamber with filtered microbes for *in situ* preservation;

(C). Filtration pump equipped at the end of the filtration system can stop the backward flow of seawater into the filter. The arrow directions in A and B indicate the liquid directions. The LIFE was undergoing underwater commissioning;

(D). The LIFE was equipped on the ROV *Faxian* along with Niskin bottles;

(E). When the ROV Faxian travelled back on the deck, the membrane in the filtration chamber was picked out immediately, quick-freezed with liquid nitrogen then stored at -80℃.

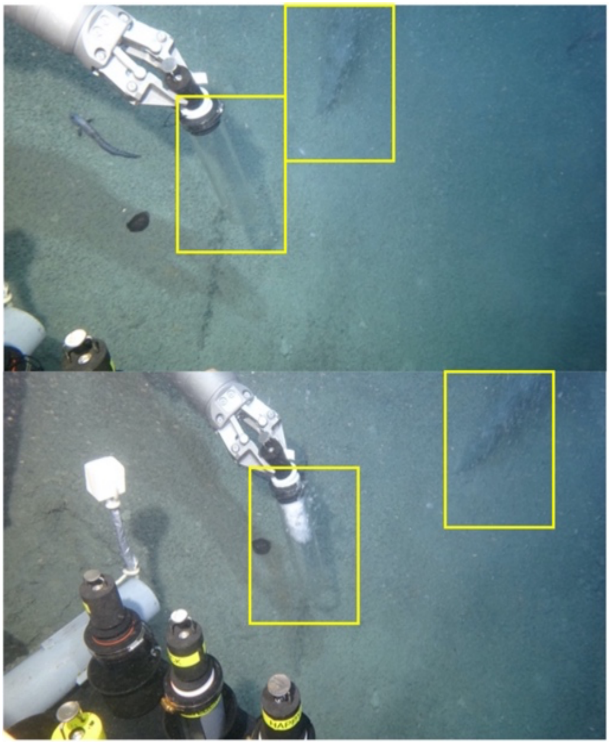

Fig. S2 Pushcore collection and accumulation of combustible ice (these pictures scattered by ROV *Faxian* Dive 252. The first picture was scattered on 12:14:40, 2021.5.28, the second was at 12:17:55, 2021.5.28. In only ~3 mins, combustible ice hydrates were accumulated at the top of pushcore in serial-shot photos).

Methane transducer is designed to measure methane concentrations, whose range is between 100nM and 50µM, with a resolution of 10nM. To ensure the integrity and precision of the instrument, when the *Faxian* ROV travelling in Lingshui seepage area, the transducer was off the charts. As a result, methane concentrations in Lingshui were not acquired. It is worth noting that methane gas bubbles were occasionally observed in Site F, while in Lingshui, methane gas was erupting continuously. And in a series of consecutively taken photographs, gas hydrate formed and accumulated at the top of pushcore (25cm in length) to a height of approximately 10cm in only ~3mins, indicating a high methane concentration in Lingshui.

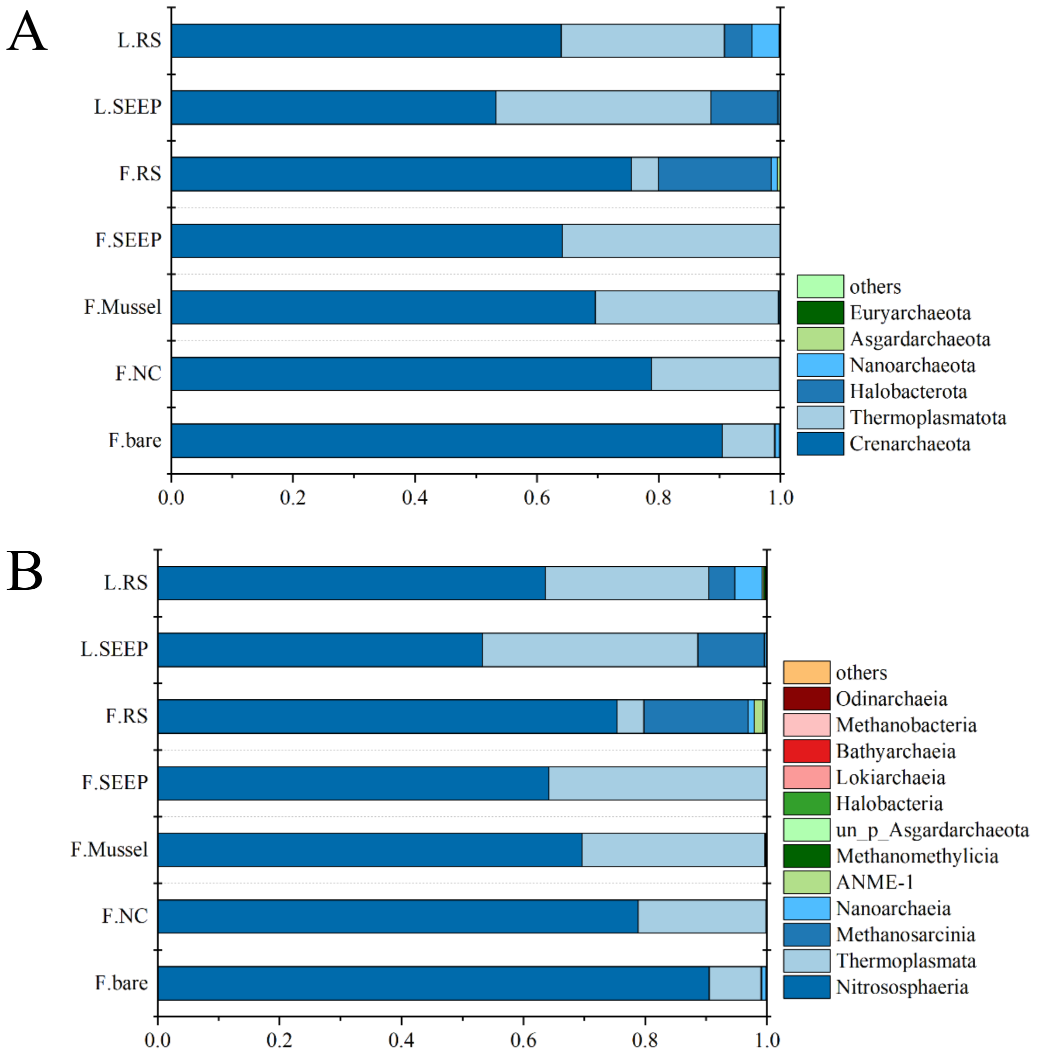

Fig. S3 Archaeal community in cold seep environment in phylum (A) and class (B) level

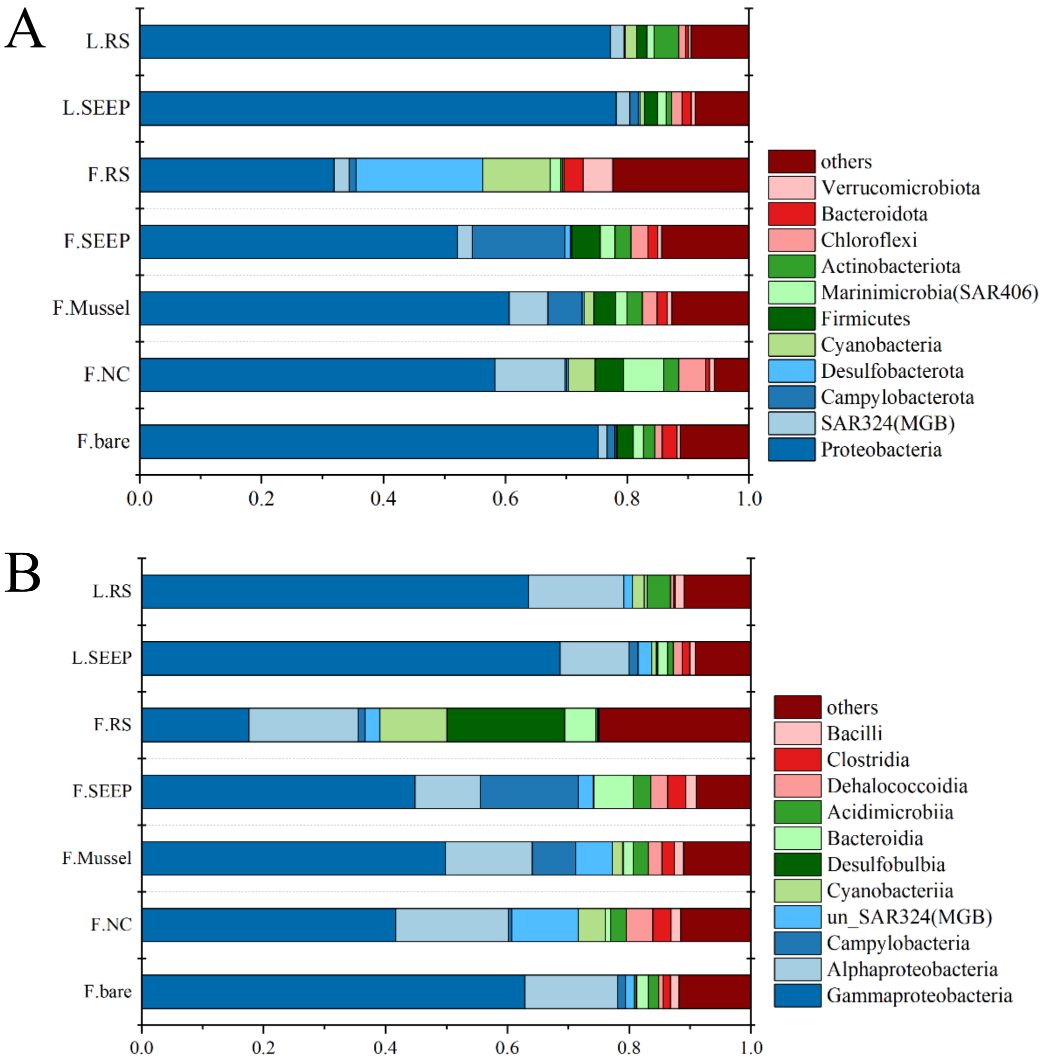

Fig. S4 Bacterial community in cold seep environment in phylum (A) and class (B) level

Tables

Table S1 archaea and bacteria 16S rRNA genes primers set procedure and protocol

genes	sequences	procedure	references
Archaeal 16S rRNA gene (288 bp)	U519F (5’- CAG YMG CCR CGG KAA HAC C) 806R (5’- GGA CTA CHV GGG TWT CTA AT)	Initially denatured at 95°C for 2 min; then amplified using 30 cycles of 95°C for 15s, 55°C for 30s, and 68°C for 45s; with final extension of 3 min at 68°C.	(Porat et al., 2010; Shehab et al., 2013)
Bacterial 16S rRNA gene (446 bp)	338F (5’- ACT CCT ACG GGA GGC AGC AG) 806R (5’- GGA CTA CHV GGG TWT CTA AT)	Initial denaturation at 95 °C for 2 min, followed by 30 cycles of denaturation at 95 °C for 30s, annealing at 53 °C for 20s, and elongation at 72 °C for 60s.	(Peiffer et al., 2013)

Table S2 Environmental characteristics of surface layer of different micro-boundary in cold seep

Sampling location	L_SEEP	L_RS	F_SEEP	F_RS	F_NC	F_Mussel	F_Bare
Sampling time	2021	2021	2020	2020	2021	2021	2021
temp (°C)	2.56	2.56	3.59	3.63	3.65	3.69	3.22
CH_4 (μM)	>50*[1]	NA	8.10	0.055	0.38	1.58	0.33
DO (mg/L)	2.4	NA	0.6-3.5	3.22	3.2	0.5-3.0	3.29
DIC (mM)	-	-	1.93	1.55	1.59	-	-
$DI^{13}C$ (‰)	-	-	-3.4	-2	-1.6	-	-
DOC (mM)			0.24	15.20	0.14		
$DO^{13}C$ (‰)	-	-	-25.63	-14.46	-28.21	-	-
NO_2^- (μM)	0.59	0.46	7.646	0.759	0.35	0.38	0.43
NO_3^- (μM)	30.2	29.73	17.365	10.608	24.2	22.32	24.31
NH_4^+ (μM)	1.93	1.95	105.374	4323.895	1.15	3.37	2.65
PO_4^{3-} (μM)	2.48	2.77	18.25	38.892	2.22	1.96	1.93
SO_4^{2-} (mM)*	28	28	28	28	28	28	28

*SO_4^{2-} Take the high sulfate concentration-28 mM- in seawater as the environment background value, which makes it the most abundant and, often, important electron acceptor at cold seeps (Joye 2020).

The DOC and inorganic nutrients possibly sourced from deep fluids associated with CH_4 seepage. The range of ammonium in Site F was ranging from 1.15 to 4.12 umol/L, while the Lingshui cold seep was relatively stable, approximately remaining at ~1.95 umol/L. The nitrite concentration of Site F was 0.32~0.43 umol/L, the nitrite concentration in Lingshui detected micro area, L_SEEP and L_RS, was higher than that of Site F, which was 0.46~0.59 umol/L. In Site F, nitrate with a high level at 20.82~24.31 umol/L, which was significantly higher than that of nitrite. The nitrate in Lingshui was about ~30 umol/L, higher than that of Site F. The total organic nitrogen (TON) in Site F (21.14~24.74 umol/L) was lower than that of Lingshui (30.19~30.79 umol/L). The concentration trend of silicate and phosphate was also consistent with that of TON, and the Site F was lower than that of Lingshui.

Table S3 Diversity Characteristics of archaeal/bacterial-16S rRNA genes

Sample Sites	Sequences (archaea/bacteria)	OTUs (archaea/bacteria)	Chao1 (archaea/bacteria)	Shannon (archaea/bacteria)	Coverage(%) (archaea/bacteria)
L_SEEP	25766/45255	175/843	213.71/922.16	4.71/6.39	99.92/99.81
L_RS	22001/47188	97/630	110.00/658.05	4.89/5.88	100.00/99.92
F_SEEP	39320/24137	125/537	119.44/1123.50	3.70/6.39	99.97/99.84
F_RS	32288/22554	284/275	311.76/275.00	4.29/5.48	99.91/100.00
F_Mussel	33881/31989	175/880	192.60/960.71	4.37/7.15	99.93/99.86
F_NC	37448/33851	118/577	124.18/624.53	3.57/6.27	99.96/99.90
F_bare	38939/36867	177/849	190.24/976.77	4.18/6.58	99.93/99.87
